# Supplementary material for: Systematic assessment of text summarization methods for biomedical literature from frequency methods to language models
Source: iScience. 2026 Sep 3;29(9):117438. doi: 10.1016/j.isci.2026.117438 (PMC13571750; doi:10.1016/j.isci.2026.117438)
Supplement: Supplementary file 1 [file mmc1.pdf]

iScience, Volume *29*

Supplemental information

Systematic assessment of text summarization methods for biomedical literature from frequency methods to language models

Fabio Baumgärtel, Enrico Bono, Lucas Fillinger, Louiza Galou, Kinga Kęska-Izworska, Samuel M. Walter, Peter Andorfer, Klaus Kratochwill, Paul Perco, and Matthias Ley

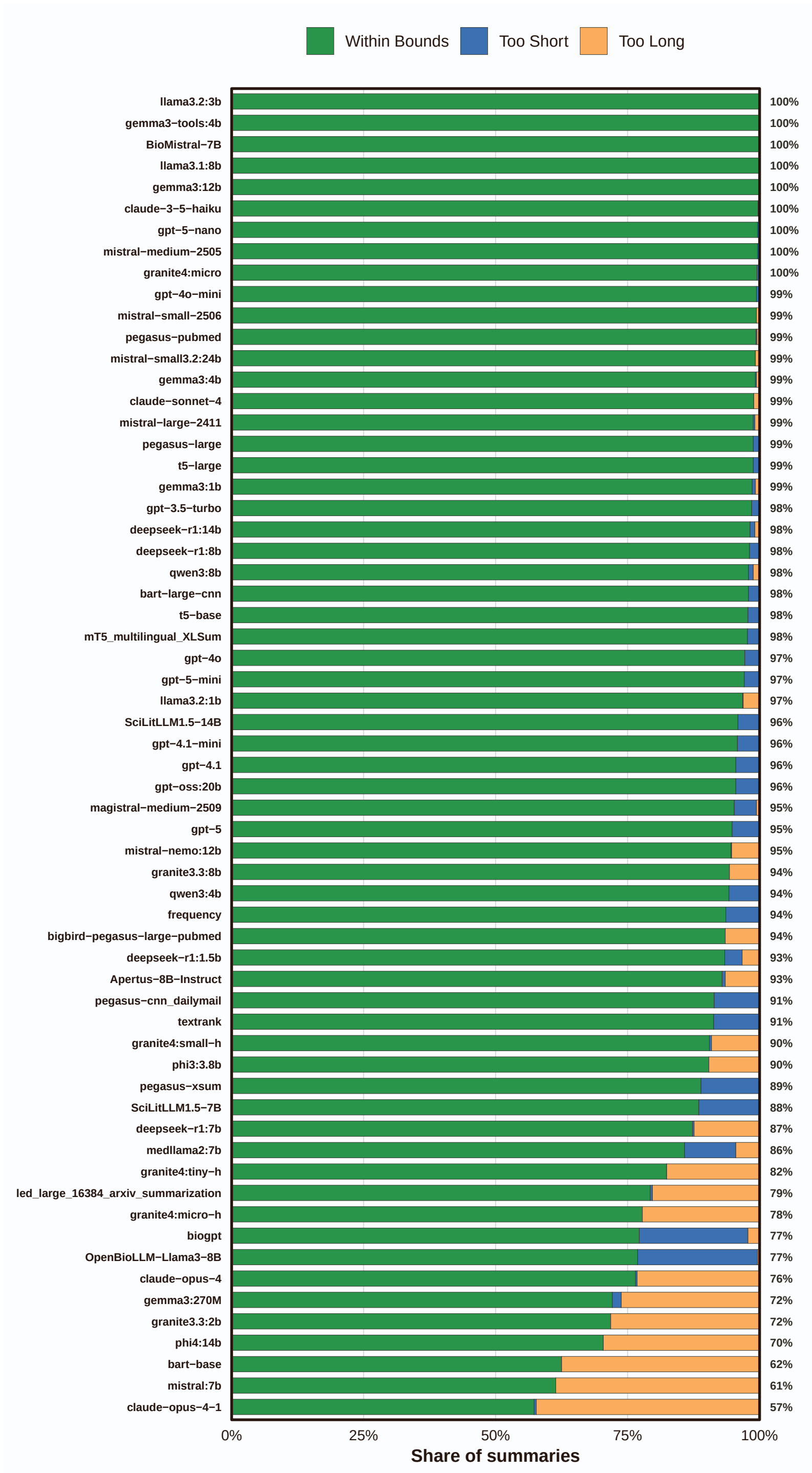

Figure S1. **Summary length compliance per method.** Proportion of generated summaries that fell within the target length of 15–100 words, were too short, or were too long, shown for each of the 62 evaluated methods. Methods are ordered by decreasing share of within-bounds summaries, with the within-bounds percentage annotated at the right of each bar.

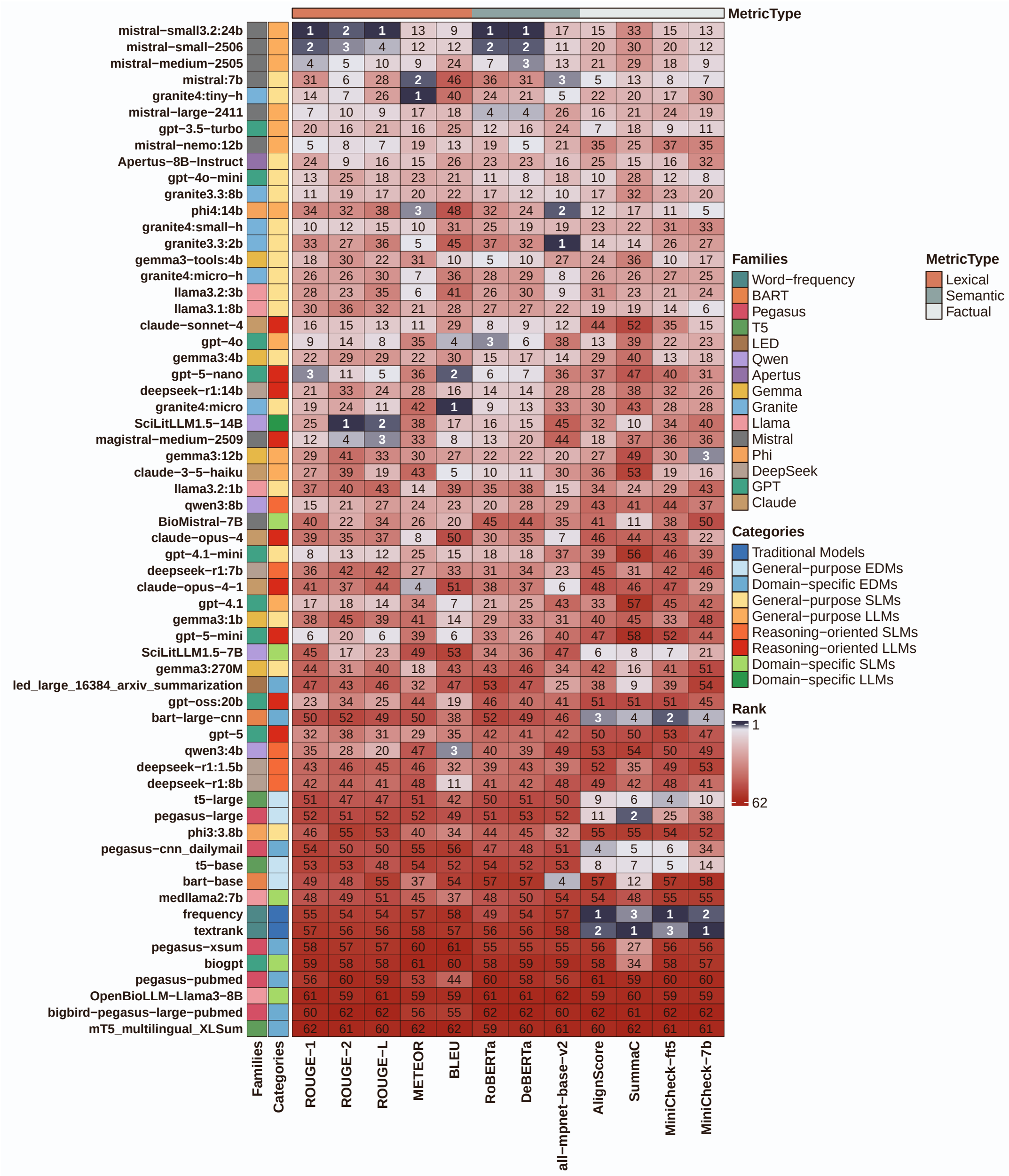

Figure S2. **Model performance across metrics.** Overview of the performance of all evaluated models across all metrics.

Table S1. **Training cutoff dates and potential overlap for all evaluated models.** The training cutoff indicates the latest point in time up to which a model may have been trained; the source of this information is given in a separate column (confirmed, community, estimated, or unknown). Potential overlap describes the percentage of benchmark articles published on or before the respective cutoff date and therefore represents the theoretical maximum share of articles that could have been present in the model's training data. Closed-source models are marked accordingly. Models are ordered by decreasing potential overlap.

**Model**	**Training cutoff**	**Source**	**Potential overlap (%)**
claude-sonnet-4-20250514 (closed-source)	2025-03	confirmed	40.2
claude-opus-4-20250514 (closed-source)	2025-03	confirmed	40.2
claude-opus-4-1-20250805 (closed-source)	2025-03	community	40.2
deepseek-r1:1.5b	2025-01	community	33.1
deepseek-r1:7b	2025-01	community	33.1
deepseek-r1:8b	2025-01	community	33.1
deepseek-r1:14b	2025-01	community	33.1
gpt-5-2025-08-07 (closed-source)	2024-10	community	24.0
gemma3:270M	2024-08	confirmed	20.3
gemma3:1b	2024-08	confirmed	20.3
gemma3:4b	2024-08	confirmed	20.3
gemma3:12b	2024-08	confirmed	20.3
PetrosStav/gemma3-tools:4b	2024-08	confirmed	20.3
phi4:14b	2024-06	confirmed	13.8
gpt-oss:20b	2024-06	community	13.8
gpt-4.1 (closed-source)	2024-06	confirmed	13.8
gpt-4.1-mini (closed-source)	2024-06	confirmed	13.8
gpt-5-nano-2025-08-07 (closed-source)	2024-05	community	11.8
gpt-5-mini-2025-08-07 (closed-source)	2024-05	community	11.8
granite3.3:2b	2024-04	estimated	10.3
granite3.3:8b	2024-04	estimated	10.3
claude-3-5-haiku-20241022 (closed-source)	2024-04	confirmed	10.3
swiss-ai/Apertus-8B-Instruct-2509	2024-03	confirmed	8.5
Uni-SMART/SciLitLLM1.5-7B	2024	estimated	6.6
Uni-SMART/SciLitLLM1.5-14B	2024	estimated	6.6
facebook/bart-large-cnn	2019	estimated	5.0
facebook/bart-base	2019	estimated	5.0
google-t5/t5-base	2019-04	estimated	5.0
google-t5/t5-large	2019-04	estimated	5.0
csebuetnlp/mT5_multilingual_XLSum	2020	estimated	5.0
google/pegasus-xsum	2020	estimated	5.0
google/pegasus-large	2020	estimated	5.0
google/pegasus-cnn_dailymail	2020	estimated	5.0
AlgorithmicResearchGroup/led_large_16384_arxiv_summarization	2020	estimated	5.0
google/pegasus-pubmed	2020	estimated	5.0
google/bigbird-pegasus-large-pubmed	2020	estimated	5.0
microsoft/biogpt	2021	estimated	5.0
aaditya/OpenBioLLM-Llama3-8B	2023-03	confirmed	5.0
BioMistral/BioMistral-7B	2023	estimated	5.0
llama3.1:8b	2023-12	confirmed	5.0
llama3.2:1b	2023-12	confirmed	5.0
llama3.2:3b	2023-12	confirmed	5.0
medllama2:7b	2022-09	confirmed	5.0
phi3:3.8b	2023-10	confirmed	5.0
gpt-3.5-turbo (closed-source)	2021-09	confirmed	5.0
gpt-4o (closed-source)	2023-10	confirmed	5.0
gpt-4o-mini (closed-source)	2023-10	confirmed	5.0
mistral:7b	—	unknown	—
mistral-nemo:12b	—	unknown	—
mistral-small3.2:24b	—	unknown	—
qwen3:4b	—	unknown	—
qwen3:8b	—	unknown	—
granite4:micro	—	unknown	—
granite4:micro-h	—	unknown	—
granite4:tiny-h	—	unknown	—
granite4:small-h	—	unknown	—
mistral-medium-2505 (closed-source)	—	unknown	—
magistral-medium-2509 (closed-source)	—	unknown	—
mistral-large-2411 (closed-source)	—	unknown	—
mistral-small-2506 (closed-source)	—	unknown	—
